# Resource-efficient Strand-seq library preparation in microliter volumes: a one-pot-style adaptation for standard laboratory infrastructure

**DOI:** 10.1016/j.mex.2026.104157

**Published:** 2026-09-10

**Authors:** Eva Benito, Ferris Jung, Catherine Stober, Patrick Hasenfeld, Maise Gomes Queiroz, Jan O. Korbel

**Affiliations:** a European Molecular Biology Laboratory (EMBL), Genome Biology Unit, Heidelberg, Germany; b European Molecular Biology Laboratory (EMBL), Genomics Core Facility, Heidelberg, Germany; c Bridging Research Division on Mechanisms of Genomic Variation and Data Science, German Cancer Research Center, Heidelberg, Germany

**Keywords:** Strand-seq, Single-cell template strand sequencing, Sustainable laboratory protocols, One-pot library preparation, Plastic waste reduction, Green chemistry

## Abstract

Strand-seq is a single-cell sequencing method that preserves directionality of DNA template strands, enabling chromosome-length haplotyping, structural variant discovery, and sister chromatid exchange mapping. The conventional, microliter-scale protocol is reliable but consumable-intensive, requiring five bead-based DNA cleanups and individual per-cell processing across a 96-well plate. A nanoliter-scale, one-pot (OP) adaptation nearly eliminates bead cleanups and plastic consumables in sub-microliter volumes on bespoke nanoarrays, but requires an acoustic dispenser, custom array hardware, and humidity-controlled dispensing not widely available. Here we describe OP-style Strand-seq, a microliter-volume adaptation retaining the central efficiency principles of the one-pot method (pooled MNase digestion, cumulative reagent addition with minimal intermediate purification, and protease-based enzyme inactivation in place of most bead cleanups) while compatible with standard 96- or 384-well plates, liquid-handling robots, and manual pipetting. Relative to the conventional protocol, OP-style Strand-seq reduces tip consumption ∼60% and eliminates three of five cleanup steps, without specialized nanodispensing hardware.

• Retains pooled MNase digestion and protease-based enzyme inactivation from the one-pot method, in microliter rather than nanoliter volumes.

• Requires two bead-based cleanups instead of five, and no specialized nanoliter dispensing hardware.

• Compatible with manual pipetting, liquid-handling robots, or acoustic dispensing in 384-well format, adoptable across diverse laboratory infrastructure.

## Graphical abstract

Schematic overview of the OP-style Strand-seq library preparation workflow. Cells are pulsed with BrdU to label newly synthesised DNA strands, nuclei are extracted and subjected to pooled micrococcal nuclease (MNase) digestion exactly as described in [1,2], and BrdU-positive nuclei are sorted by fluorescence-activated cell sorting (FACS) into individual wells containing 2 µL of release buffer. Library construction proceeds as a one-pot cascade in which three reagents are added sequentially without intermediate purification: (①) heat-mediated decrosslinking of formaldehyde-fixed chromatin, followed by protease addition for chromatin protein digestion, (②) polishing mastermix for end repair and A-tailing, and (③) adapter ligation mastermix. Following a first 0.8 × AMPure XP bead-based cleanup to remove excess adapter dimers, libraries are stained with Hoechst 33258 and exposed to UV light (365 nm, 150 s) to nick the strand that incorporated BrdU. Indexed PCR amplification is performed, followed by a second 0.8 × AMPure XP bead-based cleanup to remove unincorporated primers. Individual single-cell libraries are pooled to yield the final pooled library ready for size selection and Illumina sequencing. Icons used in the graphical abstract were sourced from BioIcons (bioicons.com) and are reproduced under the indicated licenses: cells_side_view and 96_well_plate_cells icons by Marcel Tisch (CC0); 1_5_TUBE icon by Helicase 11 (CC-BY 4.0); magnet icon adapted from u-tube icon by OpenClipart (CC0); flow-cytometer-cell-sorter icon by Derek Croote (CC0); thermalcycler-pcr icon by DBCLS (CC-BY 4.0); micropipette-multi icon by Servier (CC-BY 3.0).

**Figure:**
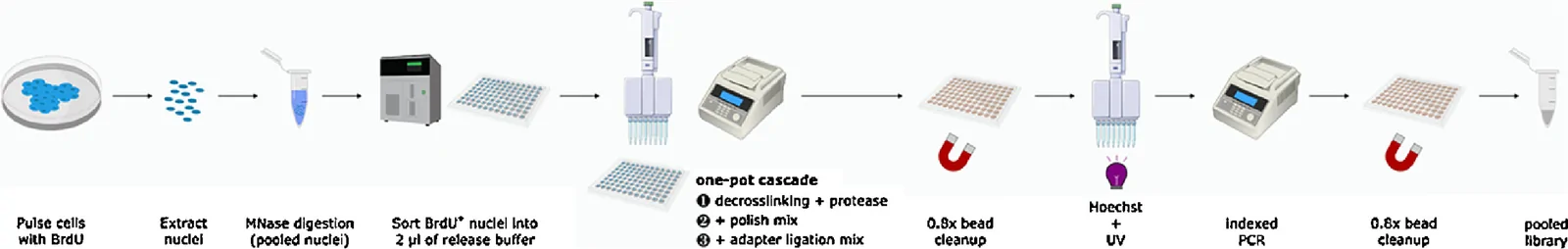

Specifications table.

**Table:** 

**Subject area**	Biochemistry, Genetics and Molecular Biology
**More specific subject area**	Single-cell genomics; single-cell template strand sequencing (Strand-seq); sustainable laboratory methods
**Name of your method**	Resource-efficient Strand-seq library preparation in microliter volumes: a one-pot-style adaptation for standard laboratory infrastructure
**Name and reference of original method**	Strand-seq: Falconer, E., et al. DNA template strand sequencing of single-cells maps genomic rearrangements at high resolution. Nat. Methods 9, 1107–1112 (2012). https://doi.org/10.1038/nmeth.2206
Scaled protocol: Sanders, A.D., et al. Single-cell template strand sequencing by Strand-seq enables the characterization of individual homologs. Nat. Protoc. 12, 1151–1176 (2017). https://doi.org/10.1038/nprot.2017.029	
One-pot nanoliter adaptation: Hanlon, V.C.T., et al. Construction of Strand-seq libraries in open nanoliter arrays. Cell Rep. Methods 2, 100150 (2022). https://doi.org/10.1016/j.crmeth.2021.100150	
**Resource availability**	No specialized dispensing hardware is required. Standard 96- or 384-well PCR plates, a multichannel pipette or standard liquid-handling robot, a magnetic separation block, a fluorescence-activated cell sorter, and a UV crosslinker (or validated UV-LED chamber) are sufficient. An optional 384-well, acoustic-dispensing (Echo) variant is described for laboratories with access to a core facility offering this instrument or a similar hardware.

## Background

Strand-seq is a medium to high-throughput single-cell genome sequencing method that exploits the semi-conservative nature of DNA replication to determine, for every chromosome in a cell, which parental DNA template strand (Watson or Crick) was inherited [1,5]. Strand-seq libraries, despite being generated at low to intermediate coverage levels, preserve directional, haplotype-resolved information lost in conventional whole-genome sequencing, enabling sister chromatid exchange mapping [1,5], the discovery and genotyping of chromosomal inversions [6], chromosome-length haplotyping [[7], [8], [9], [10]], as well as subclonal structural rearrangement discovery in normal tissues as well as in cancer cells [[11], [12], [13], [14]]. Strand-seq can be coupled with smart (adaptive-feedback) microscopy, enabling the investigation of patterns and mechanisms of chromosomal instability in real-time [15]. Furthermore, Strand-seq-based haplotype phasing can be used alongside DNA methylation detection from nanopore sequencing to assign genetic variants unambiguously not just to haplotypes but to maternally or paternally inherited homologous chromosomes [16]. The Strand-seq protocol scaled for 96-well throughput by Sanders et al [17] has become the de facto standard in the field, and is the version our laboratory at the European Molecular Biology Laboratory has been utilising routinely.

This conventional protocol is reliable but resource-intensive. Library construction proceeds through five enzymatic steps (MNase digestion, end repair, A-tailing, adapter ligation, and PCR amplification), each followed by an independent bead-based cleanup to purify DNA and remove unincorporated reagents [17]. Across a 96-well plate, this means up to five rounds of bead binding, washing, and elution per well, with a correspondingly large requirement for pipette tips and bead and ethanol volumes; reaction volumes of 10–25 µL per step further multiply consumption.

Hanlon et al [2] recently addressed this resource burden by reducing reaction volumes 500- to 1,000-fold and reformulating the workflow around three principles: (i) bulk micrococcal nuclease digestion of intact, fixed nuclei prior to sorting, rather than individual digestion per sorted cell; (ii) a one-pot scheme in which end repair/A-tailing ("polishing"), adapter ligation, Hoechst incubation and PCR proceed cumulatively in a single well without intermediate magnetic bead purification; and (iii) replacement of bead-based cleanups with thermolabile protease treatment, inactivating enzymes from the preceding step without purification. This one-pot (OP) method, dispensed in nanoliter volumes onto open, bespoke nanoarrays via an acoustic dispenser (CellenONE), reduced cost per library 6- to 16-fold and nearly eliminated plastic consumables, while improving library complexity and coverage [2].

This nanoliter format, however, requires equipment not widely available in most genomics laboratories: an acoustic or piezoelectric nanoliter dispenser, custom-fabricated open nanoarrays, and careful environmental control to limit evaporation [2]. For laboratories without this hardware, the choice has effectively been between the consumable-intensive conventional protocol and a nanoliter method that is non-trivial to implement.

We note that a high-throughput combinatorial indexing adaptation of Strand-seq, sci-L3-Strand-seq [18,19], was also recently described. sci-L3-Strand-seq achieves very low per-cell costs at scale through split-pool barcoding and linear amplification; however, it employs a substantially different library preparation chemistry based on Tn5 tagmentation rather than MNase digestion, and is primarily designed for population-scale experiments involving thousands of cells and has a reported coverage of 0.7% [18], which limits some downstream analyses. OP-style Strand-seq can be the more practical choice for laboratories running standard single-plate experiments where per-plate simplicity and automation on conventional liquid-handling robots are priorities.

We describe here OP-style Strand-seq, an adaptation applying the central logic of the one-pot method (pooled MNase digestion before sorting, cumulative one-pot processing, and protease-based enzyme inactivation in place of most bead cleanups) at microliter rather than nanoliter scale, in standard 96- or 384-well PCR plates. This sacrifices some resource savings of the true nanoliter method: microliter-scale reactions accumulate more unincorporated adapter and primer, so we retain two bead-based cleanups (after adapter ligation and after PCR) rather than eliminating cleanups entirely. In exchange, the protocol requires no nanodispensing hardware, custom arrays, or specialized humidity control: it can be executed by hand, or automated on a standard liquid-handling robot, our routine implementation. Relative to the conventional protocol, this still reduces bead-based cleanups from five to two and pipette tip consumption by approximately 60%. We position OP-style Strand-seq as a practical intermediate: more resource-efficient and thus "greener" than the field-standard conventional protocol, and more accessible than the nanoliter OP method, for laboratories seeking to reduce the environmental footprint of Strand-seq library preparation.

## Method details

**Fig. 1:**
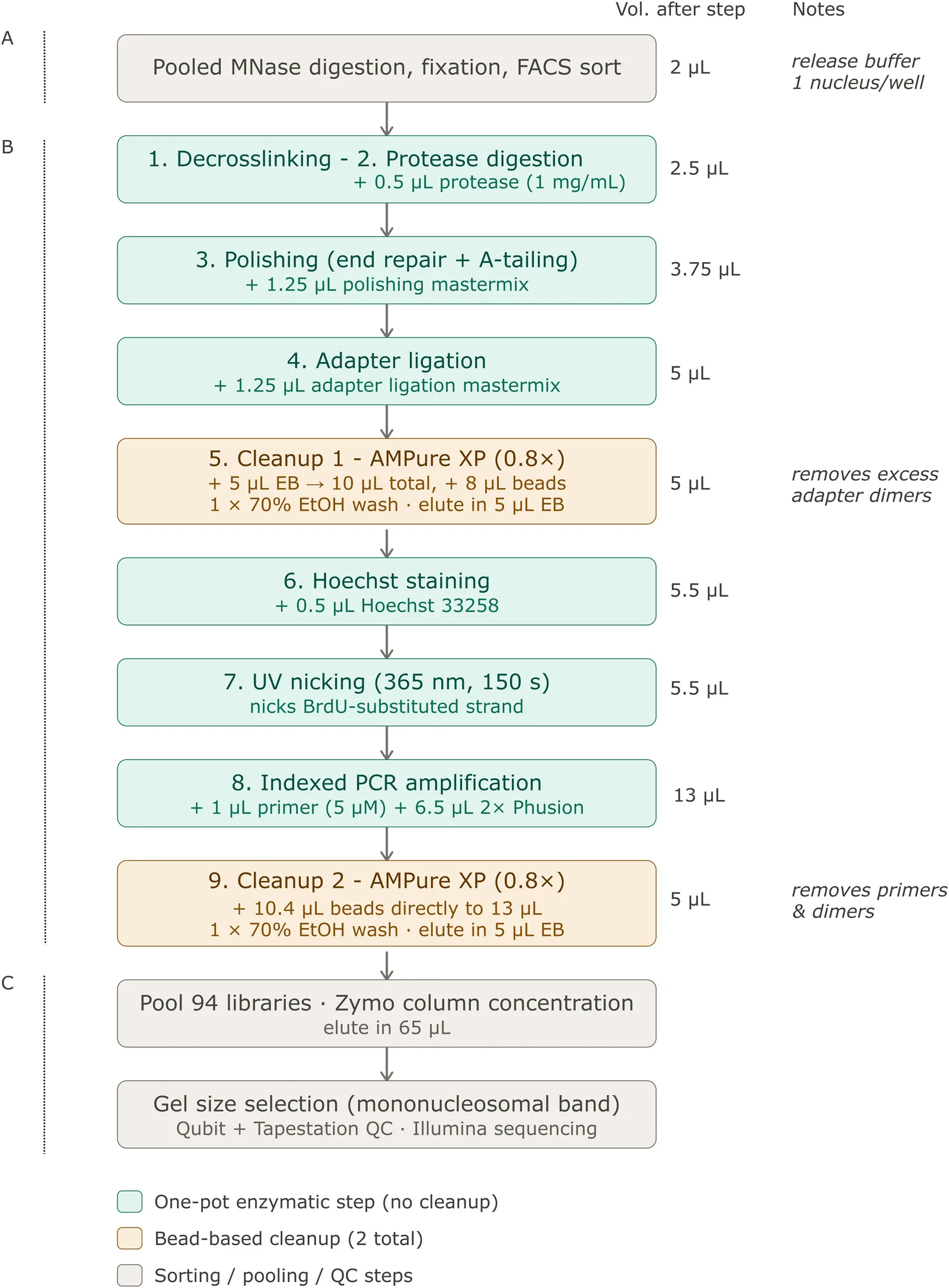
Overview of OP-style Strand-seq library construction. Schematic of the three protocol stages described in this article. (A) Nuclei preparation, pooled MNase digestion, fixation, and FACS sorting of single nuclei into individual wells, performed as previously described [2]. (B) One-pot library construction in microliter volumes: protease digestion, polishing (end repair and A-tailing), and adapter ligation proceed cumulatively without intermediate purification (green), interrupted only by two AMPure XP bead-based cleanups (orange) after adapter ligation and after PCR amplification. Cumulative reaction volume after each step is shown at right, with selected procedural notes. (C) Pooling of 94 single-cell libraries, concentration by silica-column purification, and gel-based size selection prior to Illumina sequencing.

OP-style Strand-seq follows the same overall logic as Strand-seq generally [1,2,17]: cells are cultured in the presence of the thymidine analog 5-bromo-2′-deoxyuridine (BrdU) for a single round of DNA replication, so that only the nascent DNA strand of each chromosome incorporates BrdU while the original template strand remains unsubstituted. After mitosis, daughter nuclei are isolated, and a sequencing library is constructed in which the BrdU-substituted strand is selectively nicked and excluded from PCR amplification, leaving only template-strand fragments for sequencing. The protocol below describes the three stages of our adaptation: (A) nuclei preparation, pooled MNase digestion, and single-nucleus sorting, performed as previously described by Hanlon et al. in the one pot version of the protocol [2]; (B) our modified, OP library construction in microliter volumes, the central adaptation described in this article; and (C) pooling and quality control prior to sequencing, where we also made minor, practical adaptations. See Fig. 1 for a schematic overview of the protocol.

### A. Nuclei preparation, pooled MNase digestion, and single-nucleus sorting

Nuclei preparation and pooled MNase digestion are performed without any modifications as described by Hanlon et al [2] in section “Method details, subsection A: Preparing nuclei for OP-Strand-seq libraries” and are summarized briefly here. Cells cultured in BrdU are harvested, lysed, and nuclei digested in bulk with micrococcal nuclease (MNase) to generate predominantly mononucleosomal DNA fragments, rather than digesting each nucleus individually after sorting. Nuclei are then fixed with formaldehyde. Fixed, digested nuclei are stained with Hoechst 33258. Cells that have undergone a single round of BrdU incorporation (identifiable by quenched Hoechst fluorescence relative to unsubstituted nuclei) are sorted by fluorescence-activated cell sorting (FACS). We refer users to the extensive description of logic and gating strategies in Strand-seq experiments provided in [17] and [2]. The first deviation of the method from Hanlon et al. from the protocol we are presenting is that we sort single nuclei directly into individual wells of a 96- or 384-well plate containing 2 µL of release buffer (identical in composition and recipe to that described in [2]), with one well per plate left empty as a negative (no-cell) control and one well receiving multiple (typically 100) nuclei as a positive control.

#### B1. Mastermix preparation

Prepare the following two mastermixes (Table 1 and Table 2) on ice before starting Stage B2. Both mastermixes without enzymes and iTru adapters can be aliquoted and stored at −20°C; enzymes marked with an asterisk (*) should be added fresh from stock immediately before use and not included in frozen aliquots. The adapter ligation mastermix should additionally have the iTru dual-index adapters added last, immediately before the ligation step (Stage B2, Step 4), to minimise adapter dimer formation during storage.

**Table 1:** Polishing mastermix (96-well batch, 240 µL total; dispense 1.25 µL per well).

Reagent	Catalog number	Volume (µL)
1M Tris-Acetate	Sigma-Aldrich T1503	7.2
1M K-Acetate	Merck W292001	17.97
1M Mg-Acetate	Sigma-Aldrich M5661	3.57
10% Tween-20	Biomol E108	1.92
1M DTT	Sigma-Aldrich 43816	3.57
200mM ATP	ThermoFisher J61125.06	2.4
100mM dATP	NEB N0440	3.57
10mM dNTP	NEB N0447L	1.78
T4 DNA polymerase⁎	NEB M0203L	3.22
T4 PNK⁎	NEB M0201L	10.77
Taq⁎	NEB M0273	1.99
H2O	Ambion AM9937	181.71
Total		240

⁎ Add fresh immediately before use; do not include in frozen aliquot.

**Table 2:** Adapter ligation mastermix (96-well batch, 250 µL total; dispense 1.25 µL per well).

Reagent	Catalog number	Volume (µL)
1M Tris-Acetate	Sigma-Aldrich T1503	6.24
1M K-Acetate	Merck W292001	15.57
1M Mg-Acetate	Sigma-Aldrich M5661	3.09
10% Tween-20	Biomol E108	1.58
1M DTT	Sigma-Aldrich 43816	3.09
200mM ATP	ThermoFisher J61125.06	1.99
Quick Ligase⁎	NEB M2200L	27.43
50µM iTru dual-index adapters⁎⁎	See [20]	8.23
H2O	Ambion AM9937	181.99
Total		250

⁎ Add fresh immediately before use.

⁎⁎ Add adapters last, immediately before the ligation step, to minimise adapter dimer formation.

#### B2. One-pot library construction in microliter volumes

Starting from the 2 µL of MNase-digested nucleus in release buffer (Stage A), library construction proceeds as a one-pot cascade: reagents are added cumulatively to the same well without any intermediate purification until the first cleanup step, below. All volumes refer to addition per well and assume a 96- or 384-well plate format; volumes are unchanged between manual pipetting and automated (robotic) execution. Plates are sealed during all incubation steps using adhesive aluminum foil seals (BioRad MSF1001) to prevent evaporation; in the automated implementation on the i7 Biomek liquid-handling robot, a metal lid (BioRad MSL2032) compatible with the on-deck thermocycler is used.

1. **Decrosslinking.** Incubate plate at 72C 10 min for decrosslinking as described in [2], cool to 4C.
2. **Protease digestion.** Add 0.5 µL of protease (1 mg/mL) directly to the 2 µL nucleus-containing well (final volume 2.5 µL). Incubate at 50C for 15min to digest proteins associated with the chromatin, releasing DNA fragments for downstream processing. Then incubate at 80C 15min for protease inactivation. Protease digestion as described in [2].
3. **Polishing (end repair and A-tailing).** Add 1.25 µL of polishing mastermix (final volume 3.75 µL). This single combined step repairs fragment ends and adds a 3′ adenosine overhang, as described for the OP protocol [2]. Incubate in a thermocycler at 12C 15min, 37C 15min and 85C 15min, as described in [2].
4. **Adapter ligation.** Add 1.25 µL of adapter ligation mastermix containing forked Illumina-compatible adapters (final volume 5 µL). Incubate at RT for 15min to ligate adapters to the polished fragment ends.
5. **Cleanup 1 (post-adapter-ligation).** Dilute the 5 µL reaction with 5 µL of elution buffer (EB) to bring the volume to 10 µL; this dilution step makes the subsequent bead addition practical to handle at this small scale. Add 8 µL of AMPure XP beads (0.8 × ratio relative to the 10 µL diluted sample) to selectively bind larger, adapter-ligated nucleosomal fragments while excluding smaller adapter dimer byproducts. Perform standard paramagnetic bead immobilization, one wash with 70% ethanol, and elution in 5 µL of EB. This is the first of only two bead-based cleanups in the protocol, in contrast to the conventional protocol, which requires a cleanup after each of the preceding enzymatic steps [17].
6. **Hoechst staining.** Add 0.5 µL of Hoechst 33258 (50 µg/mL) to the 5 µL eluate (final volume 5.5 µL) and incubate for 15min at RT, protected from light.
7. **UV nicking.** Expose the uncovered plate to 365 nm UV light for 150 seconds at a distance of 3 cm between the UV source and the liquid surface in the wells to nick the BrdU-substituted (nascent) strand, preventing its amplification in the subsequent PCR step. This step has been validated in our hands using two UV sources: (i) a UV crosslinker equipped with 365 nm longwave bulbs, using the irradiation conditions described previously [17]; and (ii) a custom-built UV-LED chamber (365 nm) for which photon dose has not been independently quantified in absolute units, but for which a 150-second exposure, matching the empirically determined exposure time of Hanlon et al [2], was validated to nick the BrdU-substituted strand and prevent PCR amplification, confirmed by the strand specificity of resulting sequencing data (see Protocol validation).
8. **Indexed PCR amplification.** Add 1 µL (5µM) of a uniquely indexed dual-index PCR primer (iTru dual-index PCR primer used in [2] and described more extensively in [20]), enabling multiplexed pooling of single-cell libraries and 6.5 µL of 2 × Phusion high-fidelity PCR mastermix (final volume 13 µL). Amplify using the following cycling conditions: 98°C for 30 s; 15 cycles of 98°C for 10 s, 57°C for 30 s, 72°C for 30 s; final extension at 72°C for 5 min.
9. **Cleanup 2 (post-PCR).** Add 10.4 µL AMPure XP beads directly to the 13 µL PCR product (0.8 × ratio); at this volume, no preceding dilution step is required. Perform bead immobilization, one 70% ethanol wash, and elution in 5 µL of EB. This final cleanup mostly removes unincorporated primers and primer dimers, yielding the completed, indexed single-cell library.

No wash or purification step is performed between protease digestion and adapter ligation (Steps 1–3): consistent with the one-pot principle described by Hanlon et al [2], the only interruption to the cumulative one-pot cascade in our adaptation is the bead cleanup introduced after adapter ligation (Step 5), which we find is necessary at microliter scale to remove excess adapter prior to UV treatment and PCR.

#### C. Pooling and quality control

Following the post-PCR cleanup, individually indexed single-cell libraries from the 96-well plate (94 single-nucleus libraries, separate from the one negative control well and the one positive control well) are pooled by combining equal volumes. Rather than performing repeated gel-based purification across multiple gel runs to concentrate and clean the pooled library, as is typically necessary following pooling in the conventional protocol [17], we concentrate the pool using a silica-column-based DNA clean-up kit (Zymo Research, DNA Clean & Concentrator-5, catalog number D4004) under the manufacturer's default conditions, eluting in 65 µL. This consolidates what would otherwise require several parallel gel purification runs into a single concentration step, so that only one gel needs to be run downstream for size selection.

The concentrated, pooled library is then resolved on an agarose gel, and the mononucleosomal fragment band is excised for size selection, as described previously [2,17]. The size-selected library is quantified and QC-ed by Qubit fluorometric assay and Tapestation to determine library purity and molarity prior to sequencing on an Illumina platform, following the approach described previously [2,17].

## Optional 384-well, acoustic-dispensing variant

For laboratories with access to an acoustic liquid handler (e.g., Echo; Beckman Coulter), Stage B2 above can alternatively be performed in 384-well plates at the same per-well reagent volumes described above. In this format, acoustic dispensing replaces pipetting for all reagent transfers in the one-pot cascade (Stage B2, Steps 1–4 and 6–8), so no pipette tips are consumed for these additions at all. Pipette tips are still required for the two bead-based cleanups (Stage B2, Steps 5 and 9), since bead handling, ethanol washing, and elution are performed by pipette rather than acoustic transfer. A total of six 384-tip boxes cover both cleanup steps across the full plate. On a per-library basis, this is equivalent to approximately 1.5 standard-format tip boxes per 96 libraries produced, a roughly seven-fold reduction compared to the ten 50 µL tip boxes used per 96-well plate in the standard OP-style format, while delivering four times the output. Combined with the increased throughput per plate (384 versus 96 libraries), this makes the 384-well, acoustic-dispensing variant the most consumable and especially plastic-efficient implementation of OP-style Strand-seq that we have tested, further approaching the plastic savings of the nanoliter OP-Strand-seq method while still relying on standard PCR plates rather than custom nanoarrays. Also, because the acoustic transfer volumes range from 0.5 to 5 µL, there is no need for a tightly controlled humidity environment, which is something that is not to be underestimated when handling nanoliter volumes. We note that acoustic dispensing instruments represent a substantial capital investment and are not standard equipment in most molecular biology laboratories; they are, however, relatively more accessible than nanoliter dispensing systems requiring custom open nanoarrays, and may already be available through shared genomics core facilities. We mention this variant as an optional scaling path rather than as part of the core recommended protocol, which we run routinely as described in Stages A–C in 96-well format on a standard liquid-handling robot.

## Method validation

**Fig. 2:**
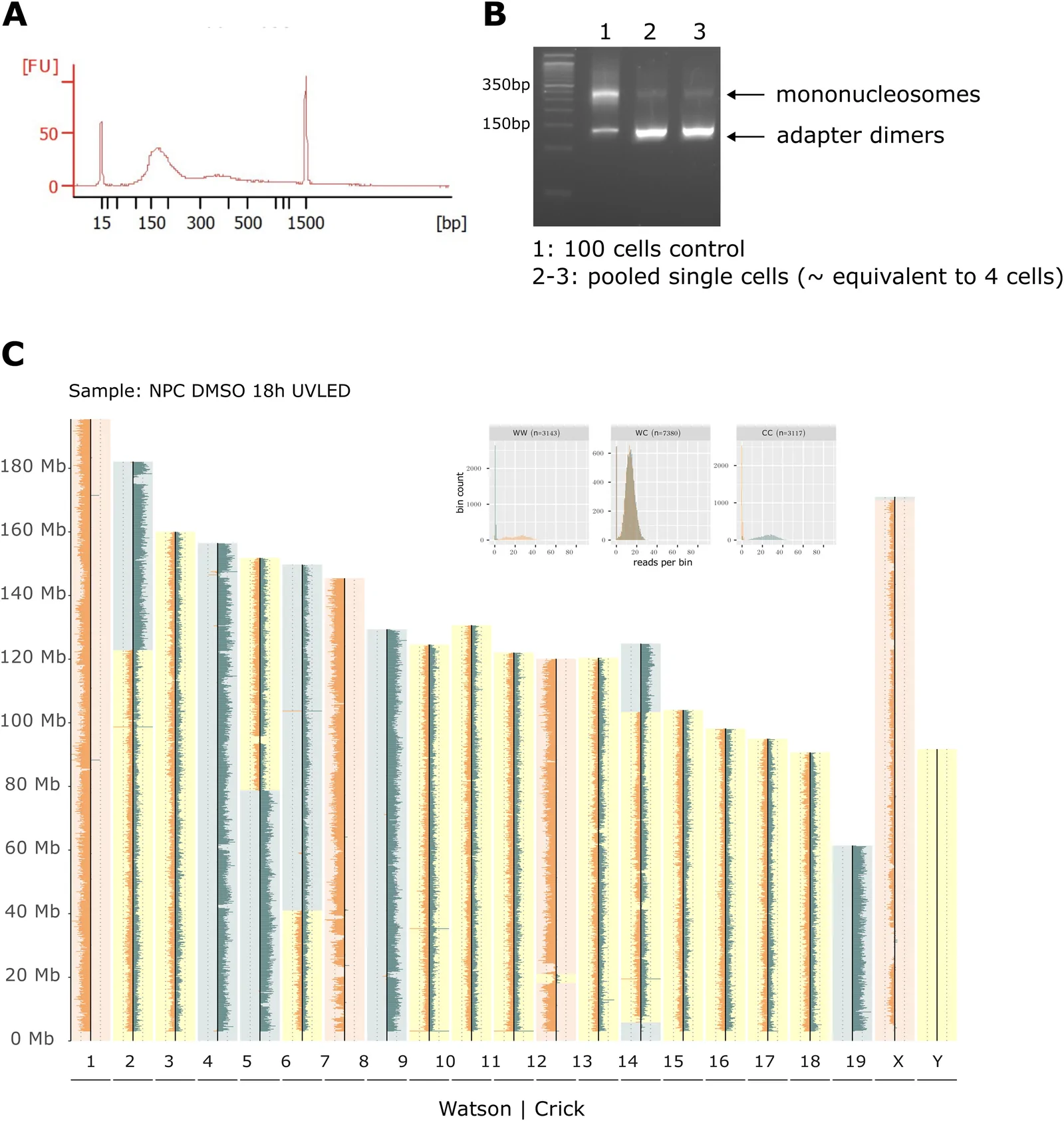
Validation of OP-style Strand-seq library quality. (A) Bioanalyzer fragment size profile of pooled, MNase-digested chromatin prior to library construction, showing the expected mononucleosomal fragment peak (∼150 bp) alongside lower (∼15 bp) and upper (∼1500 bp) marker peaks. (B) Agarose E-gel electrophoresis of completed, uncondensed libraries pooled directly without prior concentration. Lane 1: 100-cell positive control. Lanes 2–3: pooled single-cell libraries (representing approximately 4 cells per lane). Arrows indicate the mononucleosomal product band and the adapter dimer byproduct band. (C) Representative genome-wide Strand-seq ideogram from a single-cell library prepared with OP-style Strand-seq [4], generated with MosaiCatcher v2 [21], displaying Watson (orange) and Crick (teal) template strand read counts across all chromosomes. The clear, consistent partitioning of reads into Watson-only, Crick-only, or mixed (WC) chromosomal states confirms effective UV nicking of the BrdU-substituted strand and preservation of strand-specific directionality.
• Strand-specificity and genome-wide Strand-seq data: sequencing data generated from libraries prepared with this protocol show the expected Watson/Crick template strand inheritance patterns across chromosomes (Fig. 2C), confirming that UV nicking (Stage B2, Step 7), including with the custom UV-LED chamber, effectively nicks the BrdU-substituted strand and prevents its amplification during index PCR.
• Downstream use in published work: libraries generated with this protocol have supported chromosome-length haplotyping, structural variant discovery, and/or sister chromatid exchange analyses [3,4], demonstrating that library quality is sufficient for standard downstream Strand-seq analyses.
• Library complexity and genome coverage: we analyzed a set of 145 (144 for the breadth-of-coverage analysis, after excluding one cell with an unstable preseq fit) manually curated, quality-passed OP-style Strand-seq single-cell libraries from [3] using preseq [22]. Our results show adequate per-cell library complexity for downstream Strand-seq analyses, with a median depth of coverage of approximately 4.7 × 10⁻³ at a fixed sequencing effort of 20 Mb per cell, and a predicted genomic breadth of coverage reaching approximately 5% by 2 Gb of sequencing effort.

OP-style Strand-seq has been used in our laboratory to generate single-cell Strand-seq libraries for several studies [3,4]. We validated the protocol using the following lines of evidence:

• Fragment size visualisation on agarose gel and tapestation: bioanalyzer analysis of bulk, MNase-digested chromatin (Fig. 2A) and agarose gel electrophoresis of completed libraries (Fig. 2B) confirms enrichment of mononucleosomal fragments (∼150 bp insert) consistent with successful digestion and library construction.

## Resource and consumable comparison

Table 3 summarizes the reduction in pipette tip consumption associated with OP-style Strand-seq relative to the conventional protocol [17], for a single 96-well plate (96 libraries) processed by hand or by standard liquid-handling robot.

**Table 3:** Pipette tip consumption per 96-well plate, conventional Strand-seq versus OP-style Strand-seq performed on a liquid handling station, in our case the i7 Biomek robot from Beckman. The reduction reflects both the consolidation of bead-based cleanup steps (five steps in the conventional protocol versus two in OP-style Strand-seq) and the smaller number of discrete mastermix additions in the one-pot cascade.

Pipette tip size	Conventional Strand-seq [17]	OP-style Strand-seq
50 µL	27 tip boxes	10 tip boxes + 1 single tip (for iTru adapter pipetting on liquid handling station)
200 µL	2 tip boxes	1 tip box
1000 µL	0.5 tip boxes	0.25 tip boxes (17 single tips used for EtOH and EB buffer distribution + adapter ligation mastermix mixing)

Because the enzymatic formulations used in the one-pot cascade (Stage B) differ substantially in composition and staging from the discrete mastermixes used in the conventional protocol, a direct step-by-step reagent volume comparison is not very meaningful in our opinion; we therefore focus this comparison on plastic consumable usage, which is directly comparable between protocols and is a primary driver of the environmental footprint of both methods. Considering bead-based cleanups specifically, OP-style Strand-seq requires only 2 of the 5 cleanup steps used in the conventional protocol, correspondingly reducing AMPure XP bead volume, 70% ethanol wash volume, and elution buffer consumption by a similar proportion, in addition to the pipette tip savings shown in Table 3.

## Limitations

• OP-style Strand-seq does not achieve the near-complete elimination of bead-based cleanups and plastic consumables reported for the nanoliter OP-Strand-seq method [2]; two bead cleanups remain necessary at microliter scale because one-pot reactions at this volume accumulate sufficient unincorporated adapter and primer to require their removal prior to UV treatment and final library recovery.
• Library complexity of OP-style Strand-seq libraries, as assessed by preseq analysis does not reach the higher values reported for the nanoliter OP-Strand-seq method [2], consistent with the retention of two bead-based cleanup steps that are necessary at microliter scale to remove unincorporated adapter prior to UV treatment and PCR amplification. The observed coverage is nonetheless consistent with the higher end of values reported for conventional Strand-seq library preparations [17] and sufficient for downstream analyses, including structural variant discovery and haplotype phasing.
• The custom UV-LED nicking chamber described here was validated functionally, by confirming strand-specific sequencing output, rather than by independent measurement of UV photon dose in absolute units (J/m²); laboratories adopting this chamber design should similarly validate nicking efficiency empirically (e.g., by sequencing) rather than relying on exposure time alone, particularly if light source, bulb age, or chamber geometry differ from the configuration used here.
• The optional 384-well, acoustic-dispensing variant requires access to an acoustic liquid handling instrument (e.g., Echo), which represents a substantial capital cost and is typically available only through shared core facilities; it is included as an optional scaling path and is not necessary to realize the core resource savings of this protocol.
• As with the conventional and nanoliter Strand-seq protocols, this method is restricted to viable cells capable of incorporating BrdU during a single round of DNA replication and requires access to a fluorescence-activated cell sorter capable of resolving hemi-substituted nuclei.

## Ethics statements

The data and results presented in this protocol article have been generated and published previously [3,4]; the corresponding ethics statements, including any relevant institutional approvals and informed consent procedures, can be consulted in those publications.

## CRediT author statement

**Eva Benito:** conceptualization, methodology, validation, investigation, writing – original draft, writing – review and editing, visualization, project administration. **Ferris Jung**: methodology, validation. **Patrick Hasenfeld, Catherine Stober and Maise Gomes Queiroz**: validation, investigation, methodology. **Jan O. Korbel**: funding acquisition, supervision, writing – original draft, writing – review and editing.

## Declaration of generative AI and AI-assisted technologies in the manuscript preparation process

AI-assisted technologies were utilised during the manuscript preparation stage. The drafting of the text was carried out by the authors. Yet, during manuscript preparation, the author(s) used Claude (Anthropic) for the following purposes: as a writing assistant, to evaluate the pros and cons of different text structures and order of presentation, and to aid the generation of visuals that illustrate the method developed in our group, as well as differences to other methods. The author(s) carefully reviewed and edited all output of the writing assistant and take full responsibility for all of the content of the published article.

## Declaration of competing interest

The authors declare the following financial interests/personal relationships which may be considered as potential competing interests:

J.O.K. has previously disclosed a patent application (no. EP19169090) that is relevant to the use of Strand-seq for somatic structural variation analysis. The other authors declare no competing interests.

## Data Availability

No new dataset is generated by this protocol article. All data shown derive from previously published studies [3,4], where full datasets are available.
